# 3.M. Workshop: Health system barriers to vaccination in underserved communities: a tale of four countries

**DOI:** 10.1093/eurpub/ckac129.183

**Published:** 2022-10-25

**Authors:** Organised by: EUPHA-IDC, EUPHA-MIG, Chair persons: Bernadette Kumar (EUPHA-MIG), Maria Gańczak (EUPHA-IDC)

## Abstract

The literature highlights low vaccination coverage among most minority or ethnic pediatric communities in EU, including migrants. In addition to being less likely to get a vaccine, such communities may be vulnerable to developing certain infectious diseases. It is particularly important in the context of the current humanitarian crisis connected with Russia's aggression towards Ukraine. Identification of potential barriers to vaccination and system gaps is crucial to further ensure that underserved pediatric communities benefit from the same level of protection as the general population in terms of disease prevention and control, including those diseases which can be prevented by routine vaccinations. The proposed workshop moderated by well-known experts of the subject (Bernardette Kumar and Maria Ganczak) will be based on the results from qualitative data collected as a part of the 5-year RIVER-EU (Reducing Inequalities in Vaccine uptake in the European region - Engaging Underserved communities) project which tackles health system barriers among selected underserved communities living in four countries: Ukrainian migrants in Poland, females with a Turkish and Moroccan migration background in the Netherlands, marginalized Roma communities in Slovakia and migrants/refugees in Greece. This interactive workshop will provide short (5 minutes per country) comparisons of the health system barriers to vaccination against MMR (measles, mumps, rubella) and Human Papillomavirus (HPV) in children living in different underserved communities, and contrast barriers to vaccination experienced by the community members with barriers perceived by the health care professionals. International comparisons of in-depth information collected during qualitative studies (interviews and focus groups) will help to provide an increased understanding of the health system determinants of low vaccine uptake in their specific multi-factorial contexts that will vary in terms of geography, size, wealth, health sector structures, culture and vaccination law. Such qualitative research is particularly valuable regarding its potential for producing comprehensive and refinement analyses adjusted for understanding the voices of underserved communities. The session will go beyond describing those. Based on the presentations the participants will be divided into 4 groups to discuss (15 minutes) the Key Barriers; Key Facilitators to vaccination; Main lessons Learnt; Ways forward for the future to increase vaccine uptake among underserved pediatric communities to reduce the burden of vaccine preventable diseases. These 4 groups of workshop participants will be led by experts from the MEMH and IDC sections. Then, concluding remarks will be presented by each group (5 minutes per group). The final resume delivered by session chairs will end the workshop.

**Key messages:**

• The health system barriers to child vaccination among underserved communities vary among European countries, and the optimal way to adequately address them is likely to be context specific.

• The RIVER-EU project gives an opportunity to discuss lessons already learnt around the different components of the vaccination process and to pinpoint ways forward for the future to reduce the VPD.

